# Participation in Cervical and Breast Cancer Screening Among Working Women in Japan: Panel Survey Study

**DOI:** 10.2196/86885

**Published:** 2026-04-28

**Authors:** Miho Satoh, Naoko Sato

**Affiliations:** 1 Department of Nursing, Yokohama City University, 3-9 Fukuura, Kanazawa-ku, Yokohama, 2360004, Japan, 81 457872576; 2 Department of Nursing, Fukushima Medical University, Fukushima, Japan

**Keywords:** cervical cancer, breast cancer, cancer screening, personality trait, working women

## Abstract

With growing concern over high cervical and breast cancer mortality rates, this study was designed to examine the factors influencing attendance in breast and cervical cancer screening among working women in Japan by conducting a secondary analysis using nationally representative panel data from the Japanese Household Panel Survey (JHPS) and the Keio Household Panel Survey (KHPS) between 2019 and 2022, which showed that reduced screening attendance was associated with higher conscientiousness and neuroticism (possibly because of increased anxiety or fear of adverse results), suggesting that health-promoting behaviors and personality traits do not always align with preventive health actions, such as cancer screening.

## Introduction

Breast and cervical cancers have high incidence and mortality in Japan [1]. Mammography screening for breast cancer (ages 40‐69 years) aims to detect cancer early, while cervical cytology (ages >20 years) identifies human papillomavirus infection and precancerous changes to prevent cancer [2]. Both are recommended biennially and organized through municipal services under the Cancer Control Act. Japan’s breast and cervical cancer screening rates (47.4% and 43.6%) remain lower than those of other OECD (Organisation for Economic Co-operation and Development) countries [1]. With increasing workforce participation, promoting screening among working women is crucial. Working women face time constraints [3], while psychosocial factors influence health behaviors [4,5]. This study explored factors affecting screening attendance among working women, examining personality traits and risk perception.

## Methods

### Study Design and Data

This study analyzed data from the Japanese Household Panel Survey (JHPS) and the Keio Household Panel Survey (KHPS), administered by the Panel Data Research Center (PDRC) [6]. The KHPS has surveyed 4005 individuals since 2004 who were born between 1935 and 1984, while the JHPS surveyed 4000 individuals since 2009 who were born after 1927. We analyzed data from Waves 2019‐2022, including baseline demographics. The sample included women aged up to 70 years, following Japan’s breast and cervical cancer screening guidelines, which recommend screening for women older than 20 years for cervical cancer and those older than 40 years for breast cancer [2]. “Working women” were defined as female respondents employed full-time or part-time at each survey wave, with employment status as a time-varying covariate. Unemployed women were excluded from that wave’s sample. PDRC sampling weights maintained national representativeness.

### Variables

#### Dependent Variables

Breast and cervical cancer screening attendance between 2019 and 2022 was assessed. At each survey wave, participants reported whether they had undergone breast cancer screening (mammography) and cervical cancer screening (cervical cytology) within the past year, yielding a binary outcome (attended vs did not attend) for each cancer type. The dependent variable was a repeated binary outcome measured at four time points (2019, 2020, 2021, and 2022).

#### Independent Variables

Personality traits were assessed using the Japanese version of the Ten-Item Personality Inventory (TIPI-J) [7], which measures five dimensions: extraversion, agreeableness, conscientiousness, neuroticism, and openness. Each trait is evaluated on a 7-point Likert scale from 1 (“Strongly Agree”) to 7 (“Strongly Disagree”).

Risk aversion was assessed by asking respondents whether they would carry an umbrella based on rain probability in weather forecasts. A higher risk aversion ratio indicates stronger risk avoidance. This measure captures domain-general risk aversion, not cancer-specific risk perception. The JHPS/KHPS umbrella item is a validated behavioral proxy for general risk preference in Japanese panel studies [6]. While domain-general risk aversion may not perfectly reflect cancer-related health risk appraisals, research suggests that general risk preferences predict health-protective behaviors including cancer screening.

Self-rated health was measured using a 5-point scale from “Excellent” to “Poor.” Mental health was assessed using the 6-item Kessler Psychological Distress Scale [8], which evaluates depressive and anxiety symptoms over the past month.

Sociodemographic variables included age, marital status, having children or not, caregiving responsibilities, academic background, employment status, and residential area.

### Analysis

Participants completing at least two survey waves were included. A generalized linear mixed model with a binomial distribution and logit link function was used, with random intercepts for within-person correlation. For cervical cancer screening, women aged 20‐70 years were included; for breast cancer screening, women aged 40‐70 years were included, following Japanese guidelines. Wave-level missing cases were excluded while retaining completed waves, and participants with missing time-invariant covariates were removed.

Time-invariant variables were modeled as between-person predictors; time-varying covariates, at wave level. TIPI-J subscale scores (range 2‐14) were entered without standardization, with odds ratios reflecting 1-unit increments. The model incorporated demographics, personality traits, and time-varying factors using restricted pseudo-likelihood via GENLINMIXED in SPSS (version 29.0; IBM Corp). Poststratification weights from the PDRC, calibrated by age, sex, and region against census statistics, were applied owing to WEIGHT BY. GENLINMIXED’s inability to specify stratification and clustering variables for complex survey variance estimation (a noted limitation).

Analyses were performed using SPSS Statistics 29.0, with significance at *P*<.05 (two-tailed).

### Ethical Considerations

The JHPS/KHPS data used in this study were provided by the PDRC, Keio University (data provision number: 5841). This study was conducted as a secondary analysis of existing, fully deidentified panel survey data collected by the PDRC under a standing ethics framework. No new data were collected from human participants in the course of this study. In accordance with the Ethical Guidelines for Medical Research Involving Human Subjects (Ministry of Education, Culture, Sports, Science and Technology and Ministry of Health, Labour and Welfare, Japan; revised 2021) [9], secondary analyses of preexisting, anonymized datasets that were originally collected under informed consent do not require separate institutional review board approval. The original JHPS/KHPS surveys were conducted in compliance with the PDRC’s own ethical framework, and all participants provided written informed consent regarding the purpose of the study, the use of their data, assurance of anonymity, strict protection of personal information, and secondary use by the PDRC. On this basis, a separate ethics review application was not submitted for this study. The corresponding author confirms that the study was conducted in accordance with the Declaration of Helsinki.

## Results

**Figure 1.**
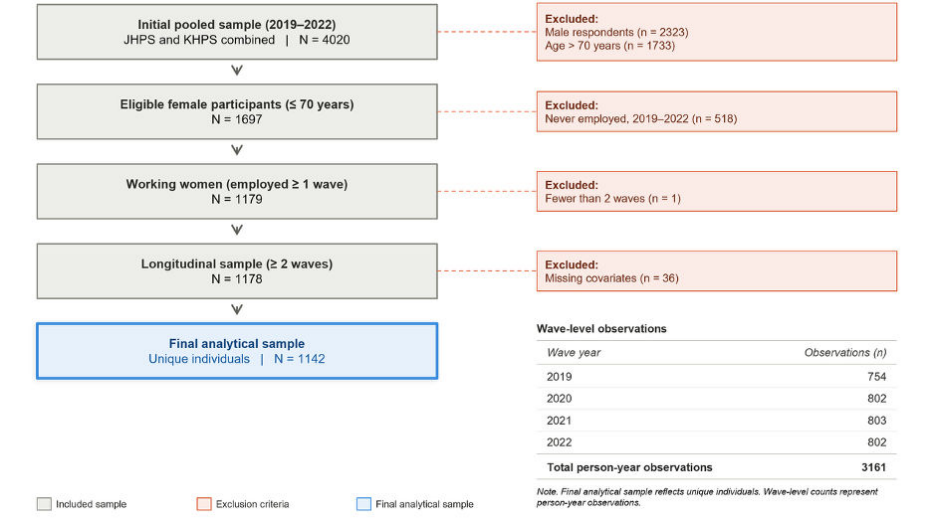
Flowchart of participant selection. JHPS: Japanese Household Panel Survey; KHPS: Keio Household Panel Survey.

Figure 1 provides an overview of the participant selection process.

Table 1 summarizes the factors associated with participation in cervical and breast cancer screenings.

**Table 1.** Factors associated with participation in cervical cancer screenings and breast cancer screenings.

		Wave 2019 (n=754)	Wave 2020 (n=802)	Wave 2021 (n=803)	Wave 2022 (n=802)	Cervical cancer screening				Breast cancer screening			
						Odds ratio	Lower limit of 95% CI	Upper limit of 95% CI	*P* value	Odds ratio	Lower limit of 95% CI	Upper limit of 95% CI	*P* value
	Cervical cancer screening participation (20‐70 years), n (%)^a^	119 (15.8)	124 (15.5)	117 (14.6)	142 (17.7)	—^b^	—	—	—	—	—	—	—
	Breast cancer screening participation (40‐70 years), n (%)	119 (17.4)	134 (18.8)	118 (15.7)	129 (17.7)	—	—	—	—	—	—	—	—
Year (reference: 2022）													
	2019	—	—	—	—	1.28	0.78	2.13	.33	1.03	0.62	1.71	.92
	2020	—	—	—	—	1.01	0.64	1.60	.95	0.91	0.57	1.45	.69
	2021	—	—	—	—	1.37	0.87	2.21	.19	1.15	0.72	1.84	.57
Age group^c^, mean (SD)^d^													
	29‐39 years	89 (11.8)	84 (10.5)	48 (6.0)	73 (9.1)	—	—	—	—	—	—	—	—
	40‐49 years	269 (35.7)	253 (31.5)	222 (27.6)	242 (30.2)	0.74	0.34	1.60	.45	—	—	—	—
	50‐59 years	253 (33.6)	285 (35.5)	307 (38.2)	288 (35.9)	1.08	0.48	2.40	.86	1.48	0.91	2.40	.11
	60‐70 years	143 (19)	180 (22.4)	226 (28.1)	199 (24.8)	1.10	0.46	2.62	.84	1.38	0.76	2.53	.29
Extraversion, mean (SD)		8.60 (2.55)	—	—	—	0.92	0.84	1.01	.09	0.92	0.84	1.01	.09
Openness, mean (SD		7.59 (2.17)	—	—	—	1.04	0.93	1.16	.51	1.02	0.91	1.14	.72
Conscientiousness, mean (SD)		7.79 (2.16)	—	—	—	0.88	0.79	0.98	.02	0.86	0.77	0.97	.01
Neuroticism, mean (SD)		8.08 (2.16)	—	—	—	0.88	0.78	0.99	.04	0.87	0.77	0.98	.02
Agreeableness, mean (SD)		9.89 (1.82)	—	—	—	1.01	0.90	1.15	.84	0.96	0.85	1.09	.57
Risk aversion, mean (SD)		47.78 (16.73)	46.94 (16.55)	48.21 (16.96)	47.68 (16.32)	1.01	1.00	1.02	.21	1.01	1.00	1.02	.15
Mental health, mean (SD)		5.65 (5.15)	5.49 (5.00)	4.91 (4.52)	5.04 (4.73)	0.98	0.93	1.03	.40	0.99	0.95	1.04	.74
Self-rated health, mean (SD)		3.35 (0.89)	3.41 (0.91)	3.47 (0.89)	3.41 (0.87)	1.03	0.80	1.31	.83	1.01	0.80	1.29	.91
Marital status, n (%)													
	Reference: unmarried	201 (26.7)	220 (27.4)	218 (27.1)	219 (27.3)	—	—	—	—	—	—	—	—
	Married	553 (73.3)	582 (72.6)	585 (72.9)	583 (72.7)	0.76	0.43	1.34	.34	0.72	0.40	1.28	.27
Child status, n (%)													
	Reference: ≥15 years old	538 (71.4)	565 (70.4)	565 (70.4)	565 (70.4)	—	—	—	—	—	—	—	—
	None	120 (15.9)	136 (17)	140 (17.4)	137 (17.1)	0.90	0.44	1.83	.77	0.78	0.38	1.60	.50
	<15 years old	96 (12.7)	101 (12.6)	98 (12.2)	100 (12.5)	0.75	0.39	1.44	.39	0.64	0.33	1.25	.19
Caregiver role status, n (%)													
	Reference: Yes	138 (18.3)	155 (19.3)	157 (19.6)	151 (18.8)	—	—	—	—	—	—	—	—
	No	616 (81.7)	647 (80.7)	646 (80.4)	651 (81.2)	1.02	0.58	1.78	.95	1.18	0.68	2.03	.56
Education background, n (%)													
	Reference: university/graduate school	313 (46.8)	331 (46.7)	328 (45.9)	334 (46.8)	—	—	—	—	—	—	—	—
	Junior/senior high school	130 (19.4)	239 (33.7)	242 (33.9)	235 (33)	0.71	0.39	1.31	.28	0.60	0.33	1.10	.10
	Junior/technical college	226 (33.8)	139 (19.6)	144 (20.2)	144 (20.2)	0.85	0.52	1.38	.50	0.74	0.45	1.21	.23
Employment status, n (%)													
	Reference: temporary	531 (70.4)	552 (68.8)	551 (68.6)	554 (69.1)	—	—	—	—	—	—	—	—
	Permanent	223 (29.6)	250 (31.2)	252 (31.4)	248 (30.9)	0.96	0.59	1.55	.86	1.03	0.64	1.66	.91
Region of residence, n (%)													
	Reference: towns or villages^e^	62 (8.2)	65 (8.1)	66 (8.2)	67 (8.4)	—	—	—	—	—	—	—	—
	Government-designated city^f^	453 (60.1)	482 (60.1)	483 (60.1)	487 (60.7)	1.44	0.67	3.06	.35	1.59	0.76	3.33	.22
	City^g^	239 (31.7)	255 (31.8)	254 (31.6)	248 (30.9)	1.47	0.72	2.98	.29	2.24	1.11	4.53	.02

a For categorical variables.

b Not applicable.

c Reference category for age differed by outcome: 29‐39 years for cervical cancer screening and 40‐49 years for breast cancer screening.

d For continuous variables.

e Population is <50,000.

f Population is ≥500,000.

g Population is ≥50,000 to <500,000.

For cervical cancer screening, women with higher conscientiousness (odds ratio [OR] 0.88, 95% CI 0.79‐0.98; *P*=.02) and neuroticism (OR 0.88, 95% CI, 0.78‐0.99; *P*=.04) showed lower attendance likelihood.

For breast cancer screening, women with higher conscientiousness (OR 0.86, 95% CI 0.77‐0.97; *P*=.01) and neuroticism (OR 0.87, 95% CI 0.77‐0.98; *P*=.02) showed reduced attendance.

## Discussion

This study showed that women with high conscientiousness and neuroticism were less likely to participate in cancer screening. While conscientious individuals typically show strong self-discipline linked to preventive health behaviors [4,10], our findings revealed increased conscientiousness correlated with reduced screening uptake. Japanese women in demanding roles may prioritize work and family over preventive health care. Higher neuroticism may increase anxiety about diagnoses, discouraging screening [11], though both require investigation.

Study limitations include potential recall and social desirability biases from self-administered questionnaires, selection bias from listwise exclusion of missing data, general rather than cancer-specific risk aversion measures, and lack of health care trust and access barrier data. Using women in their 30s as a reference group may be misleading, since mammography is not recommended for this age in Japan. Future research should examine structural and psychosocial factors affecting screening.

## Abbreviations

JHPS: Japanese Household Panel Survey
KHPS: Keio Household Panel Survey
OECD: Organisation for Economic Co-operation and Development
OR: odds ratio
PDRC: Panel Data Research Center
TIPI-J: Japanese version of the Ten-Item Personality Inventory
